# Blended learning in nursing pharmacology: elevating cognitive skills, engagement and academic outcomes

**DOI:** 10.3389/fphar.2024.1361415

**Published:** 2024-02-22

**Authors:** Hadar Arien-Zakay

**Affiliations:** The Faculty of Medicine, School of Pharmacy, Institute for Drug Research, The Hebrew University of Jerusalem, Jerusalem, Israel

**Keywords:** pharmacology education, question-based learning, Bloom’s taxonomy, pre-clinical education, student’s engagement

## Abstract

Pharmacological education is crucial for healthcare professionals to safely manage medications and reduce errors. Traditional lecture-based learning (LBL) often struggles to address this complexity, whereas newer methods, such as flipped classrooms and problem-based learning, yield mixed results, particularly in pre-clinical contexts, owing to students’ limited experience. Our nursing pharmacology course under LBL recorded a high failure rate of 37.8% and marginal passing scores across five cohorts (*n* = 849 students). An analysis using Bloom’s taxonomy revealed significant gaps in higher-order cognitive skills. As a remedy, the course was transformed into a novel blended learning format that integrated question-based learning (QBL) to enhance critical thinking across all cognitive levels. This model blends asynchronous and synchronous learning, is tailored to individual needs in large classes, and fosters continuous, student-centric learning. The redesign markedly decreased the failure rate by approximately 2.8-fold and increased the average grade by 11.8 points among 426 students. It notably improved the pass rates in advanced cognitive categories, such as “Evaluate” and “Create” by 19.0% and 24.2%, respectively. Additionally, the blended course showed increased student engagement, reflecting a dynamic and effective learning environment that significantly elevated participation and academic outcomes at all cognitive levels. This study demonstrated the profound impact of blended learning in pharmacology. By integrating QBL with various teaching methods, it surpasses traditional lecture-based limitations, enhancing engagement and understanding of complex topics by nursing students. Notable improvements in foundational and advanced learning suggest its broader application in health professionals’ education, effectively equipping students for clinical pharmacology challenges.

## 1 Introduction

Pharmacology education aims to enhance the competency of health professionals in medication management and patient safety. Mastering its complexities remains a challenge for students (Engels, 2018; Preston et al., 2019; Rubaiy, 2021). While active learning shows promise, the effectiveness of traditional lecture-based learning (LBL) versus new methods is still under examination. Advanced pedagogies such as flipped classrooms (FC) and problem-based learning (PBL) have been suggested as effective and satisfactory methodologies for enhancing self-learning skills and competencies in many aspects of medical education (Rui et al., 2017; Hew and Lo, 2018; Stentoft, 2019; Youhasan et al., 2021). However, in the field of pharmacology, as well as in other fields studied in the pre-clinical years, data are still controversial or point to a gap between the desired improvement in students’ experience and the achieved learning outcomes necessary for further clinical training (Evans et al., 2019; Trullàs et al., 2022; Xiao et al., 2023). For pre-clinical students, mastering pharmacology via FC and PBL methods can be challenging, as they often do not have sufficient background knowledge to navigate complex topics such as drug interactions and side effects. This lack of experience may result in learning gaps, as students may neglect crucial pharmacological details in favor of more attractive subjects, underscoring the limitations of FC and PBL as effective teaching methods for pharmacology (Shanley, 2007; Karpa and Vrana, 2013). Additionally, the feasibility of implementing such advanced teaching methods, especially in larger classes, raises concerns (Pastirik, 2006). These approaches require increased human resources and ongoing training for their effective implementation. Faculty members face increased workloads, potential confrontations with students, and technological challenges (Pastirik, 2006; Trullàs et al., 2022).

Our department, which is responsible for pharmacological and pharmacotherapeutic instruction of nursing, pharmacy, and medical students, faces significant challenges. The complex yet vital curriculum designed to prepare participants for practical training is difficult to modify. Moreover, increasing student numbers due to clinician shortages and increasing cultural diversity (Admi et al., 2018) add to the complexity of addressing individual academic needs. Unlike a more gradual approach in pharmacy and medical studies, nursing students tackle intensive pharmacology courses in their second year, which may affect their readiness to grasp the complexities of pharmacological processes. Among those students, we witnessed high failure rates in a primary pharmacology course and an apparent deficiency in fundamental knowledge and abilities, leading to course retakes, postponement of clinical mentorship, and, in some cases, discontinuation of studies.

To overcome these challenges, we have developed a new blended course model that aims to foster a continuous, individualized learning environment. Although constrained by resource limitations and large class sizes, this approach focuses on identifying and overcoming learning outcome deficiencies through question-based learning (QBL). We used Bloom’s taxonomy (Giffin, 2002) as a guide to categorize question types, design the curriculum and assessments, and emphasize the development of higher-order thinking skills. Using this strategy, students were encouraged to actively seek answers and cultivate their critical thinking and problem-solving abilities (Freeman et al., 2014; Torralba and Doo, 2020) but were directed to focus on all cognitive levels throughout the course, ensuring a structured sequence in their learning process.

The course structure utilized a hybrid format that combined asynchronous online activities with synchronous in-person sessions to enhance students’ engagement and learning outcomes (Morton et al., 2016). This methodology aims to ensure balanced focus across all cognitive levels, from basic knowledge to advanced problem-solving skills. Finally, the learning outcomes of the new blended course were evaluated and found to significantly enhance both fundamental and advanced learning, according to Bloom’s taxonomy scaling. This result underscores the potential of blended learning to effectively address educational challenges in pharmacology, suggesting its broader applicability in professional health education.

## 2 Materials and methods

### 2.1 Primary pharmacology course for nursing students

The primary pre-clinical pharmacology course included the following modules: basic principles, fundamentals of pharmacokinetics, pharmacodynamics and pharmacotherapeutics, autonomic drugs, cardiovascular-Renal drugs, drugs with significant effects on smooth muscle, drugs used for blood disease treatment, drugs influencing glucose balance, anti-inflammatory drugs, and pulmonary pharmacology.

Subsequent topics were addressed either within smaller specialized pharmacology courses or integrated within other pre-clinical modules.

### 2.2 The LBL course structure

The lecture-based learning (LBL) format spanned a 14-week semester, totaling 56 h. Each week, four academic hours were dedicated exclusively to in-person lectures. Starting in 2016, this LBL approach, catering to approximately 170 students per cohort, was complemented by a problem-based learning (PBL) workshop that focused on cardiovascular-renal system pharmacology. This workshop featured small group discussions, with 10–12 students per group, delving into pharmacological treatments through case studies, all facilitated by a teaching assistant. Workshop preparations entailed attending in-person lectures and studying pertinent course book chapters, along with drug information condensed into a workshop booklet. The workshop grade, which contributed to the final course score, included an initial test to measure students’ basic understanding and a concluding test to assess their ability to apply this knowledge to clinical case scenarios. These tests comprised of multiple-choice and open-ended questions.

The course’s final exam encompassed all topics presented during the term, assessing the pharmacological skills and knowledge necessary for students’ upcoming clinical training in hospitals in the following semester. This exam comprised 40 multiple-choice questions, in a randomized order, with five options each. Students were required to correctly answer at least 24 of these questions (minimum grade 60).

### 2.3 The blended course structure

In 2022, a renewed course model was launched and has since been adopted by two student cohorts with 216 and 210 students in 2022 and 2023, respectively. The course, curated primarily by the coordinator, maintained the original LBL timeline but integrated a blended learning approach, melding the flipped classroom paradigm with question-based learning (QBL). The content was structured hierarchically, breaking the syllabus down into distinct units, each with different pharmacological dimensions (Table 1).

**TABLE 1:** Structure and content of the blended pharmacology course.

**Learning Division I: Basic Principles**	
Learning Units:	- *Introduction to Pharmacology*
	- *Fundamentals in Pharmacokinetics*
	- *Fundamentals in Pharmacodynamics and Pharmacotherapeutics*
	- *Key Types of Drug Interactions, Adverse Reactions and Toxicity*
	- *Receptors as Drug Targets*
**Learning Division II: Autonomic Nervous System Drugs**	
Learning Units:	- *Introduction to Autonomic Pharmacology*
	- *Cholinergic Drugs*
	- *Anti-Cholinergic Drugs*
	- *Adrenergic Drugs*
	- *Anti-Adrenergic Drugs*
**Learning Division III: Cardiovascular-Renal Drugs**	
Learning Units:	- *Anti-Hypertensive Agents*
	- *Diuretic Agents*
	- *Vasodilators and the Treatment of Angina Pectoris*
	- *Agents Used in Heart Failure*
	- *Agents Used in Cardiac Arrhythmias*
	- *Drug Therapy for Dyslipidemias*
**Learning Division IV: Hemostasis, Glucose Balance, and Immunity System Drugs**	
Learning Units:	- *Drugs Used in Disorders of Coagulation*
	- *Pharmacotherapy of Diabetes Mellitus*
	- *Anti-Inflammatory, Anti-Allergy, and Immunosuppressant Drugs*
	- *Pulmonary Pharmacotherapy*

Progression began when the learners accessed advanced pre-recorded videos and live synchronous sessions. Subsequently, they participated in online quizzes. Asynchronous online participation was facilitated via a dedicated Moodle™ platform, which served as the primary Learning Management System (LMS). This platform displayed an array of tools: video sessions concluded with quizzes and supported by specialized Q&A and news forums. Employing PowerPoint^®^ (Microsoft Corporation, Redmond, Washington) and Panopto™ (Panopto: Seattle, Washington), lectures were constructed and uploaded as concise videos to the course site using Panopto™ technology. The online quizzes created within the LMS incorporated immediate post-completion feedback to further the learning curve.

Following to 4–6 h of asynchronous online activity, students participated in 2-h synchronous, in-person discussions with the course faculty. These sessions, held in a hall room, mandated full attendance as specified in the course guidelines. During these meetings, students engaged in peer learning, interacting with questions projected on the screen, which were subsequently discussed under the faculty’s guidance.

Notably, the cardiovascular system pharmacology workshops remained unchanged from the LBL format. Likewise, the structure of the concluding examination was held in class and remained consistent with the previous versions.

### 2.4 Questionnaires

The study utilized a self-administered electronic questionnaire developed according to practice guidelines for surveys (Artino et al., 2014). Data were collected anonymously at the end of the course and before the final examination. Although the native language of the nursing student population varied (i.e., Hebrew, Arabic, Russian, etc.), all the students were proficient in Hebrew, including reading and writing. Participants were provided with information about the voluntary nature of their participation and assurances of anonymity and confidentiality with no impact on their assessment. The questionnaires, focusing on overall course satisfaction via a graded scale, collected responses from five LBL cohorts (*n* = 328) and two blended course cohorts (*n* = 133), using the same format for consistency. Additionally, they featured open-ended sections for students to elaborate on the positive and negative aspects of the course. This study was approved by the Ethics Committee of Hebrew University (number 05122023).

### 2.5 Data analysis

Data collected from the two cohorts of the new blended course structure (*n* = 426 students) were compared with data from 5 years of cohorts from the previous LBL course (*n* = 849 students). The LBL cohort sizes for 2016–2020 were 160, 170, 184, 169, and 166 students, respectively. Data from 2021 were omitted because of the disruptions caused by the COVID-19 pandemic.

Data were analyzed using GraphPad Prism (Boston, MA, United States) statistical software and are presented as mean ± SD. Frequency distributions, with numbers and percentages for all variables, were produced. *t*-test and One-way ANOVA statistical tests were used for comparison between categorical variables; *p*-value < 0.05 was considered to be significant.

## 3 Results

### 3.1 Pharmacology education in LBL structure leads to insufficient learning outcomes

**FIGURE 1:**
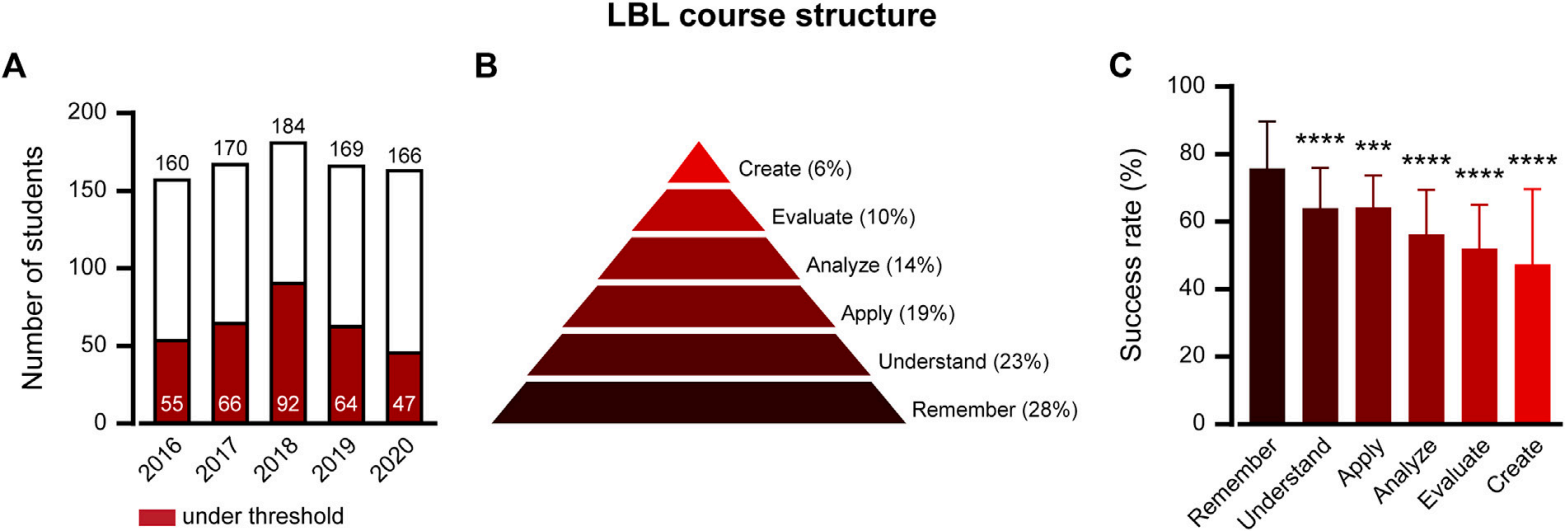
The LBL pharmacology course structure resulted in low-performance trends in learning outcomes. **(A)** Student success rate in LBL Course Structure final exams, 2016–2020: A comparison of students scoring below the pass threshold (red bars) to the total number of students who sat for the exam (white bars). **(B)** Percentile of LBL course questions in 2016–2020 final exams divided by Bloom’s taxonomy cognitive levels. **(C)** Success rates by cognitive question levels. Percentages reflect the mean ± SD of students correctly answering the test questions at each cognitive level. Each level was compared with “Remember”. ***p ≤ 0.001; ****p ≤ 0.0001; one way ANOVA followed by multiple comparison test.

For more than 25 years, our pharmacology course for nursing students in pre-clinical training was structured around in-class lecture-based learning (LBL). In the past decade, problem-based learning (PBL) workshops have complemented LBL. A review of the course’s academic outcomes identified a recurring challenge: A substantial percentage of students did not meet the required standards on their final exams (Figure 1A). On average, across five cohorts, 37.8% ± 7.9% of students did not pass the course. An in-depth analysis of the grade distribution revealed a troubling trend: the average final exam score across these cohorts was 64.6 ± 16.0, alarmingly close to the minimum passing score of 60. This suggests that a large segment of students was not just underperforming, but barely reaching the threshold of competence.

To better comprehend the gaps in student learning, we applied Bloom’s taxonomy (Giffin, 2002) and methods from Kim et al. (2012) to analyze pharmacology exam questions. Each question was categorized into the taxonomy’s six cognitive levels:

1) ***Remember***: Recognizing basic drug knowledge; e.g., Identifying drug names.
2) ***Understand***: Interpreting pharmacological concepts; e.g., Explaining how a drug’s mechanism of action results in its therapeutic effects.
3) ***Apply***: Using theories in new situations: e.g., Selecting a drug, based on a patient’s condition.
4) ***Analyze***: Differentiating among drug effects; e.g., Determining which of a patient’s several drugs could cause a noted side effect.
5) ***Evaluate***: Judging therapeutic approaches; E.g., Ranking drug choices by considering patient factors like age or kidney function.
6) ***Create:*** Designing new solutions; e.g., Design a new treatment plan to reduce potential drug interactions.

An example of the graduated questions is presented in Supplementary Table S1.

The final exam questions from the five LBL cohorts were then classified (Figure 1B) and the success rate in each category was calculated (Figure 1C). Questions at the “Remember” level recorded the highest success, with 75.7% ± 14.0% of students answering correctly. By contrast, the success rate for questions at all other cognitive levels was significantly lower. The “Understand” and “Apply” categories presented challenges, with correct response rates of 64.1% ± 11.9% and 64.1% ± 9.4%, respectively. For the “Analyze” category, roughly 56.3% ± 13.2% of students could successfully break down complex pharmacological scenarios. The “Evaluate” category shows only 51.7% ± 13.0% of students able to integrate and act on multiple factors in patient-based cases. Finally, the demanding “Create” level presented the most significant challenges, with just 47.1% ± 22.1% of students being able to construct solutions from various case details.

Student feedback from end-of-course surveys offers insights into the course’s strengths and areas for improvement. Comments were grouped into themes for analysis. The positive aspects (104 comments) included the course’s relevance (39%), its organization (12%), and the quality of teaching (23%), with the workshop being particularly appreciated as beneficial for learning (26%). However, improvement suggestions (121 comments) pointed to the course’s difficulty (32%), teaching style concerns (14%), and organizational issues (12%). Notably, 41% of the students recommended a more practice-oriented approach to enhance content retention and understanding.

### 3.2 Developing the blended course structure to improve higher cognitive learning outcomes

In response to the identified academic deficits, the pharmacology course was substantially restructured into a new blended structure. The central aim was to immerse students in critical thinking exercises across all cognitive tiers of Bloom’s taxonomy. This comprehensive approach was designed to equip students with rigorous analytical skills that are imperative for safe and adept administration of drugs in clinical scenarios.

The course was redesigned with a hierarchical structure segmented into learning divisions, each tailored to cover specific pharmacological aspects (Table 1). This ensured the focused learning of one set of concepts before transitioning to the next. Within these divisions, the content was meticulously segmented into concise learning units, each containing a scaffolded learning experience that progressively elevated students from foundational knowledge retention to sophisticated problem-solving abilities. The learning journey unfolds with increasing intricacy, starting with knowledge acquisition using synchronically in-person or asynchronically recorded videos. Thereafter, students took online quizzes, reinforcing their understanding and tapping into all levels of critical thinking skills, proportionally divided in similarity to Figure 1B. Altogether, 130 online quizzes were distributed in each learning unit as follows: remember 27% ± 2.9%, understand 22% ± 2.4%, apply 18% ± 2.1%, analyze 16% ± 1.4%, evaluate 10% ± 1.1%, and create 7% ± 2.3%. Following the practice of online questions in each learning unit, students were gathered for synchronous face-to-face discussions. The objective of these sessions was to deepen the understanding of and pinpoint challenging areas informed by student performance on online quizzes. These discussions further explore how slight shifts in the pharmacological situation can dramatically alter the scenario, resulting in significant clinical consequences. This pedagogical method encourages students to elevate to higher levels of applying, analyzing, evaluating, and creating critical thinking.

In the cardiovascular renal drug division, students encounter the same complex problem-solving exercises integral to the LBL course structure. Addressed in small groups, these exercises were designed to promote in-depth discussions, propelling students toward the analysis, evaluation, and creation of the stages of Bloom’s taxonomy (Shawahna, 2021).

Finally, the course culminates in an online mock examination that allows students to practice across a spectrum of cognitive complexities within all learning divisions.

### 3.3 Blended course structure elevates learning outcomes across all cognitive levels

**FIGURE 2:**
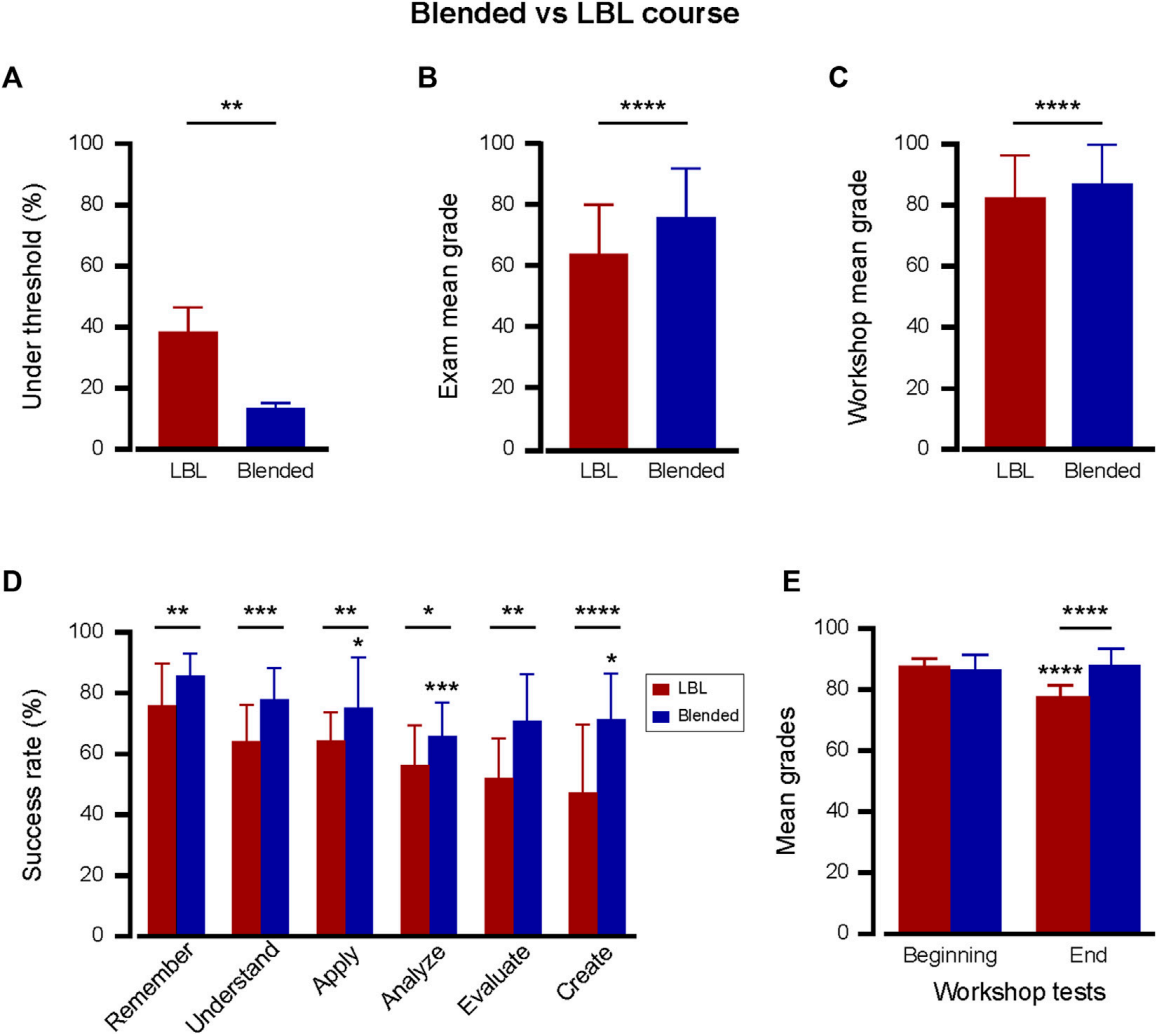
The Blended course structure dramatically enhanced learning outcomes. Superior testing outcomes with blended (*n* = 426; two cohorts; blue bars) vs. LBL (*n* = 849; five cohorts; red bars) pharmacology course structures. **(A)** Relative percentile of students scoring below the pass threshold of 60 in the final exam. The rates are the average of the cohorts in each course structure. **p < 0.01; Two-tailed unpaired *t*-test. **(B, C)** Mean ± SD grades in the final course exam **(B)** and in the cardiovascular-renal workshop test **(C)**. ****p < 0.0001; Two-tailed unpaired *t*-test. **(D)** Comparative success rates across cognitive levels between blended (*n* = 80 questions in two cohorts, blue bars) and LBL (*n* = 200 questions in five cohorts, red bars) structures. *p < 0.05; **p < 0.01; ***p < 0.001; ****p < 0.0001; Two-tailed unpaired *t*-test between each cognitive level. In the blended, each level was also compared with “Remember.” *p ≤ 0.05; ***p < 0.001; one way ANOVA followed by multiple comparison test. **(E)** Beginning- and end-cardiovascular-renal workshop test comparisons for blended (*n* = 6 tests for each test type across two cohorts) and LBL (*n* = 15 tests for each test type across five cohorts) approaches. The beginning-test covered lower cognitive skills (Remembering, Understanding, Applying), while the end-test evaluated higher cognitive skills in drug-based patient care (Analyzing, Evaluating, Creating). In LBL, ****p < 0.0001 in beginning-vs. end-workshop grades; Between LBL and blended, ****p < 0.0001 in end-workshop grades. In the blended test, no statistically significant differences were observed between the beginning- and end-workshop tests.

As the pharmacology nursing course underwent a transformational shift from the classical LBL method to the question-based blended approach, a significant improvement in academic performance was evident (Figure 2). The examination results revealed a marked contrast in the failure rates between the instructional formats. While the LBL format had a high average failure rate of 37.9% ± 7.9%, the blended method substantially lowered it to 13.6% ± 1.5%, indicating a reduction of 24% (compare Figure 2A; Figure 1A). In line with the failure rate, the blended course structure delivered an improved average grade of 76.4 ± 16.0. The observed difference in the mean score between the two methods was 11.8, indicating a statistically significant improvement in the blended course structure (Figure 2B). Furthermore, a histogram depicting exam grade distribution for both the LBL and blended course structures further illustrates this positive shift, showing a notable increase in the frequency of higher grades among students in the blended format compared with the LBL approach (Supplementary Figure S1). An improvement was also observed in small-group activity. Examining the cardiovascular-renal workshop test scores, the LBL structure yielded a mean of 82.2 ± 14.1. In contrast, the blended format surpassed this with an average of 86.6 ± 13.2, marking a significant enhancement in scores by 4.4 points (Figure 2C). These results strongly suggest that the blended format increases the learning outcomes of the course. However, a possible explanation for this data is an increase in student satisfaction with the new format. To test this, average student satisfaction data derived from anonymous surveys were examined. The analysis showed no statistically significant change with an average of 8.3 ± 0.7 vs. 7.8 ± 1.3 (out of 10.1) for blended and LBL, respectively.

Given the observed academic advantages of the blended course approach in the final exams, we sought to understand its impact on learning outcomes categorized by cognitive level. Therefore, the success rates across cognitive levels in the blended course exams were compared with those presented in Figure 1C for the LBL format. In every cognitive category, the blended course demonstrated a significant upward trend in student success rates in the blended course structure as compared to the LBL (Figure 2D). As in the LBL format, questions at the “Remember” level recorded the highest success, with 85.5% ± 7.3% of students answering correctly. In contrast to LBL, a less dramatic decrease in the success rate of questions at all other cognitive levels was observed for the blended format. A notable enhancement was observed in the higher cognitive realms of “Evaluate” and “Create,” where the blended format demonstrated a leap of 19.0% and 24.2% points, respectively. These findings underscore the blended courses’ proficiency in fostering advanced cognitive skills.

The juxtaposition of beginning- and end-cardiovascular-renal workshop test outcomes in both blended and LBL formats offered additional insights (Figure 2E). While the beginning-test was designed to evaluate lower levels of cognitive skills (Remembering, Understanding, Applying), the end-test was used to assess advanced cognitive abilities concerning drug-based patient care (Analyzing, Evaluating, Creating). A pronounced increase in end-workshop test scores of 11 was observed for the blended structure compared to the LBL. This substantiates the enhanced capacity of students in the blended course to navigate the complexity of higher-order pharmacological problem-solving.

In summary, not only does the blended course format offer superior overall academic results but it also notably enhances outcomes across all cognitive domains, especially in the intricate realms of evaluation and creation. This synergistic effect confirms the blended course’s efficacy in creating a holistic, well-rounded learning environment in pharmacology.

### 3.4 The blended structure fosters a deeply engaging learning atmosphere

Finally, to test whether the blended course generated a better learning environment, the dynamics of student engagement was the next focus.

**FIGURE 3:**
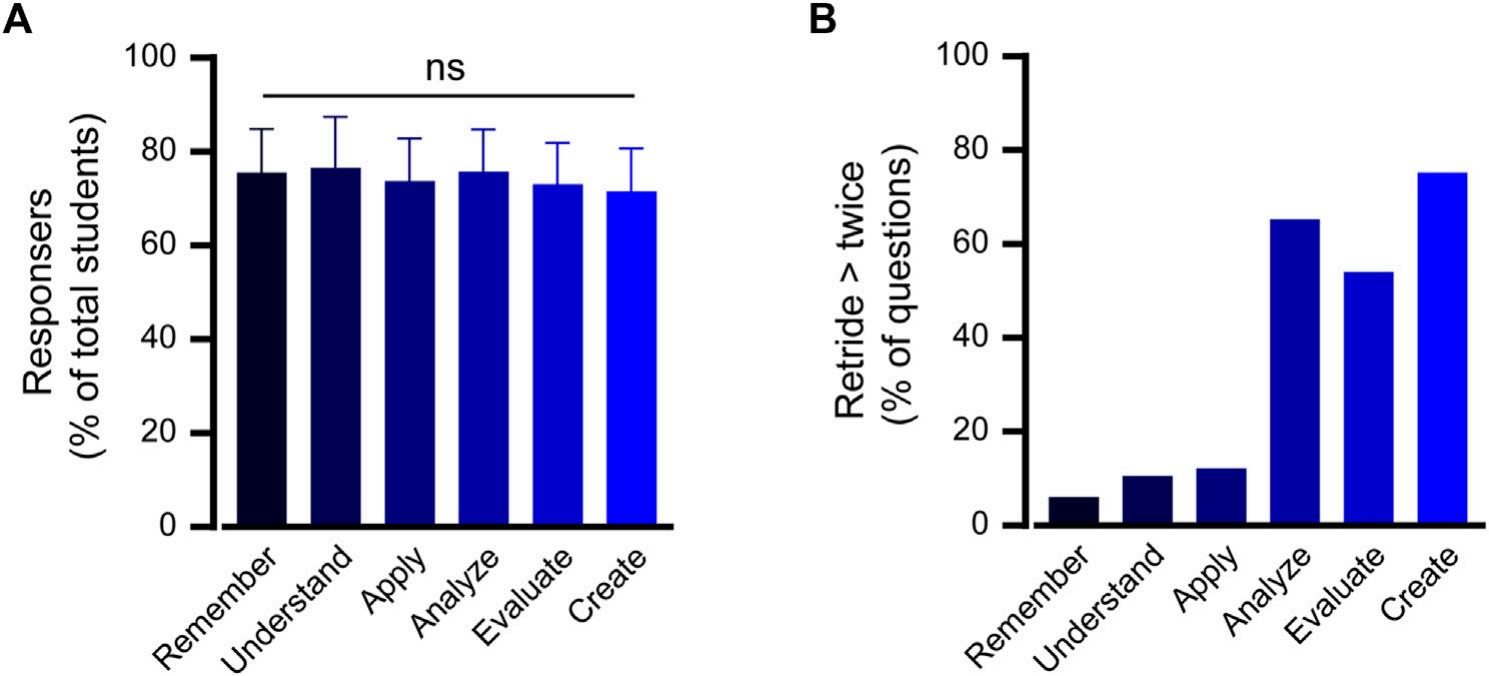
High engagement pattern in the blended course structure. **(A)** The participation rate in online quizzes is presented as a percentage of students enrolled in the blended course. The data are sorted according to cognitive learning level of question type and the average frequency of question attempts across all learning units is depicted. One way ANOVA analysis followed by a multiple comparison test indicated no statistically significant difference compared with the “Remember” level. **(B)** Fraction of online questions students attempted more than twice. This metric showcases the percentage of questions that students retried two or more times, broken down by cognitive learning level outcomes.

Students exhibited substantial engagement with online quizzes that encompassed the spectrum of cognitive levels defined by Bloom’s Taxonomy (Figure 3A). A consistent trend emerged across all six cognitive domains, with an average participation rate of 74.1% ± 1.9% of registered students. Such uniformity indicates that the online platform was effective in engaging students irrespective of the cognitive difficulty of the questions or the pharmacological topic they addressed.

An important metric of perseverance and determination in mastering a topic is willingness to reattempt challenging questions. The data revealed a segment of students who revisited specific questions twice or more. Such retrials were also categorized based on cognitive learning outcomes, thereby providing a lens into areas where students might have faced initial difficulties, but displayed resilience to enhance their understanding (Figure 3B). The data show that most repeated questions were related to advanced cognitive levels of analysis, evaluation, and creation, which involve decision-making considering multiple factors in a patient case (Kim et al., 2012).

Essentially, the blended course appeared to have successfully fostered a culture of active participation and iterative learning. Students not only actively engaged with the content but also displayed a commendable willingness to persevere through challenging materials.

## 4 Discussion

In this study, transitioning of a nursing pharmacology course from traditional LBL to blended learning significantly improved student outcomes, with a notable increase in average grades by 11.8 points and an approximately 2.8-fold decrease in the failure rate. Pass rates improved in all cognitive skills, notably in “Evaluate” and “Create,” with increases of 19.0% and 24.2%. This approach improved student engagement, emphasizing the efficacy of integrating question-based learning with diverse teaching methods.

The study introduces a new blended learning model that aims to fulfill the potential of flipped classroom (FC) and problem-based learning (PBL) methods to enhance pharmacological skills among pre-clinical students. This approach addresses the gap between the theoretical promises of these methods and their actual, somewhat limited effectiveness, as documented in previous research. This structure, which relies on practicing questions across all cognitive levels within each learning unit, reaffirms the potential of adaptive learning strategies to foster a thorough understanding, while also emphasizing the importance of simultaneously reinforcing lower cognitive levels to establish a foundational knowledge base. As evidenced by the significant reduction in failure rates and improvement in average scores, the blended course format appears superior to traditional LBL complementary to the PBL method. These results highlight the ability of the blended course to provide a conducive learning environment that addresses the full spectrum of cognitive abilities, ultimately resulting in superior academic outcomes. The ability to guide students to a higher cognitive plateau, such as Evaluate and Create, not only strengthens their foundational knowledge but also enhances their capacity for critical thinking and application in complex real-world scenarios. Of note, although not examined in this study, integrating elements of the blended format into the LBL format–such as online practice questions and student-led peer sessions with feedback–could potentially enhance motivation and learning outcomes with minimal ongoing costs or faculty effort.

Considering nurses’ significant challenges, high stakes in patient care (Bigi and Bocci, 2017), and the variability in feelings of unpreparedness for drug use (Preston et al., 2019), developing effective pharmacological education is vital for enhancing patient safety and nurses’ confidence in drug administration (Prochnow et al., 2022). A recent comprehensive study evaluating various educational strategies to enhance drug competency suggests that the FC, PBL, and Team- and Case-Based Learning could improve pharmacology education (Xiao et al., 2023). However, these studies primarily focused on medical and pharmacy students, with less than 20% involving nursing students. Therefore, the current literature reveals the need for more research concerning these methods in nursing education, as well as other approaches, such as blended learning.

The blended learning structure presents the notable benefit of fostering an engaging and motivational learning environment. It employs adaptive learning strategies structured to encourage learner motivation and engagement (Cecchini-Estrada and Méndez-Giménez, 2017; Torralba and Doo, 2020). This is evident from the high levels of student participation across various cognitive domains and their readiness to re-engage with difficult content, thus nurturing a proactive learning culture, skill development, and in-depth understanding of the subject matter, which is an environment conducive to nurturing essential skills for lifelong learning (Artino et al., 2012; Ross et al., 2022). Moreover, given its digital components, the blended approach is inherently scalable, allowing institutions to cater to larger student cohorts without diluting the quality of education. During the COVID-19 pandemic, the shift toward digital learning methods likely had a positive impact on the successful implementation of the blended pharmacology course. In this period students (Doluweera et al., 2023) and faculty (Alqahtani et al., 2023) became more adept at engaging with online educational tools, potentially setting a precedent for future digital education advancements (Zhang et al., 2023). Finally, with technological advancements, it is conceivable that the blended format could integrate even more sophisticated tools, such as artificial intelligence-powered tutors or smartphone apps, to further enhance the learning experience (Sahanaa and Mishra, 2018; Civaner et al., 2022; Nagi et al., 2023).

This study had several limitations. A significant pedagogical shift may involve an initial resistance or skepticism from educators and students. Although a substantial portion of student comments on the LBL structure called for a more practice-based approach, this did not translate into a significant change in student satisfaction scores when comparing the LBL and blended methods. This consistency in satisfaction levels indicates that, while students accepted the change, there is still potential to enhance their overall satisfaction (Ramnanan and Pound, 2017). The strength of the evidence in this study is supported by the large number of participants, consistency in course content and instructors, and stable curriculum and student background during the evaluation years. Expanding the implementation of the blended method to different pharmacological and pre-clinical settings could strengthen the validation of the results. Our department, for example, provides pharmacology courses tailored for nursing, pharmacy, and medical students. This study focused on a nursing primary pharmacology course, which has historically had the lowest success rate among pharmacology courses. The gap in success rates may be partially explained by the unique structure and timing of the nursing curriculum, which introduces complex pharmacological concepts earlier in studies, compared to a gradual approach in pharmacy and medical courses. In such situations, where curriculum modifications are restricted, implementing alternative teaching methods such as blended learning may be particularly effective in enhancing student comprehension and success rates. Thus, the effectiveness of the blended learning approach in pharmacology education depends on the needs of the course, and is not necessarily suitable for every educational structure.

Moreover, the success of the blended approach relies on technological infrastructure and access. In today’s world, this enables participants to be engaged in their own schedules, adapting their learning to the time and place that best suit their needs (Sormunen et al., 2020). Additionally, the structure of a blended course, which allows for competencies and skills acquisition through online engagement, offers increased adaptability in the face of unforeseen events, such as the COVID-19 pandemic. However, this approach assumes that all students and teachers have access to the digital tools. Institutions that consider this model must ensure robust support systems to overcome potential technological barriers (Söderlund et al., 2023).

In conclusion, this study underscores the transformative potential of a blended course structure in pharmacological education. It is evident that to cater to contemporary academic demands and the multifaceted cognitive needs of students, more adaptive and comprehensive methods such as the blended approach are beneficial and essential. As academia continues to evolve, pedagogical tools should ensure that we deliver education that is both contemporary and effective.

## Data Availability

The original contributions presented in the study are included in the article/Supplementary Material, further inquiries can be directed to the corresponding author.
