# Supplementary material for: Blended learning in nursing pharmacology: elevating cognitive skills, engagement and academic outcomes
Source: Front Pharmacol. 2024 Feb 22;15:1361415. doi: 10.3389/fphar.2024.1361415 (PMC10917888; doi:10.3389/fphar.2024.1361415)
Supplement: Supplementary file 1 [file Table1.pdf]

Supplementary Material

Table 1. Applying Bloom's taxonomy to pharmacology exam questions: graduated questions on ACE inhibitors as an example (translated from Hebrew)

1) Remember: Recognizing basic drug knowledge; e.g., Identifying drug names.

Which of the following is an ACE inhibitor?

A. Enalapril
B. Valsartan
C. Atenolol
D. Hydrochlorothiazide
E. Amlodipine

2) Understand: Interpreting pharmacological concepts; e.g., Explaining how a drug's mechanism of action results in its therapeutic effects.

Which of the following best explains how ACE inhibitors reduce arterial blood pressure?

A. They decrease vasoconstriction by lowering the synthesis rate of angiotensin II
B. They lead to vasodilation by enhancing the breakdown of bradykinin
C. They reduce blood volume by increasing sodium and water excretion They
D. They enhance vasodilation by blocking calcium channels in vascular smooth muscle
E. They lower cardiac output by decreasing the sympathetic nervous system activity

3) Apply: Using theories in new situations: e.g., Selecting a drug, based on a patient's condition.

A 58-year-old patient with type 2 diabetes, has elevated blood pressure. Which medication provides renal protective benefits and would be most appropriate for blood pressure control in this patient?

A. ACE inhibitor
B. Beta-adrenergic blocker
C. Calcium channel blocker
D. Aldosterone receptor antagonist
E. Alpha-adrenergic blocker

4) Analyze: Differentiating among drug effects; e.g., Determining which of a patient's several drugs could cause a noted side effect.

A 69-year-old patient with a past medical history of congestive heart failure, type II diabetes, and coronary artery disease presents for follow-up. The patient is taking Spironolactone, Enalapril, Atorvastatin, Metformin and Empagliflozin. The patient is reporting a persistent dry cough. Which medication is most likely causing the cough?

A. Enalapril
B. Metformin
C. Spironolactone
D. Empagliflozin
E. Atorvastatin

5) Evaluate: Judging therapeutic approaches; E.g., Ranking drug choices by considering patient factors like age or kidney function.

For a hypertensive patient with a history of chronic heart failure and kidney disease, which medication would be the least suitable?

A. Amlodipine
B. Losartan
C. Lisinopril
D. Furosemide
E. Metoprolol

6) Create: Designing new solutions; e.g., Design a new treatment plan to reduce potential drug interactions.

A patient with hypertension controlled by Ramipril develops hyperkalemia. What modification in the medication regimen could address the hyperkalemia while maintaining effective blood pressure management?

A. Add Hydrochlorothiazide
B. Add Spironolactone
C. Increase Ramipril dosage
D. Switch to Losartan
E. Add Amlodipine

* For current presentation, the correct answer is A. ACE – Angiotensin converting enzyme.
