# Supplementary material for: Blended learning in nursing pharmacology: elevating cognitive skills, engagement and academic outcomes
Source: Front Pharmacol. 2024 Feb 22;15:1361415. doi: 10.3389/fphar.2024.1361415 (PMC10917888; doi:10.3389/fphar.2024.1361415)

Supplementary Material

Figure 1. Positive shifts in exam scores under the blended course structure. The histogram illustrates the exam grade distribution with blended (n=424; two cohorts; blue bars) vs. LBL (n=859; five cohorts; red bars) pharmacology course structures. The X-axis represents the range of exam grades in gaps of 5 points. The Y-axis displays the percentile frequency of students in each grade category relative to the total number of students in each course structure.

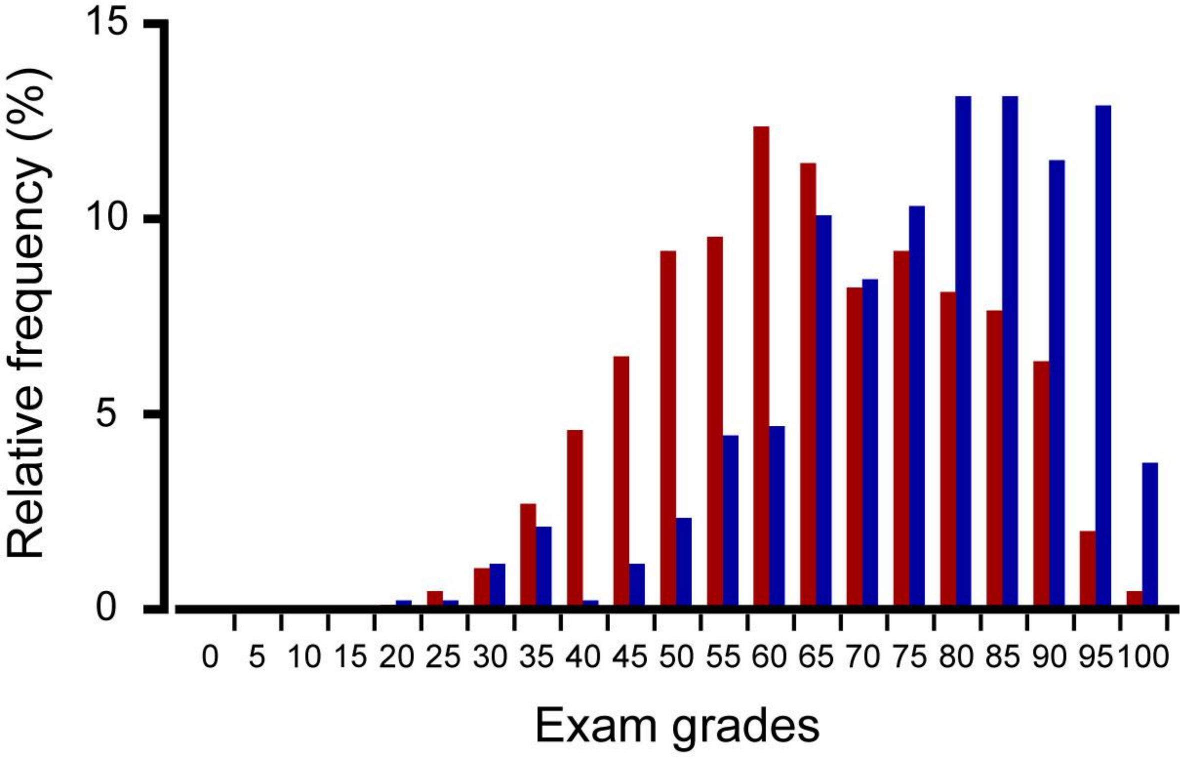

Supplement: Supplementary file 2 [file Image1.pdf]
